# Generating, evaluating, endorsing, and implementing malevolent creativity: a malevolent idea journey

**DOI:** 10.3389/fpsyg.2025.1695259

**Published:** 2025-12-04

**Authors:** Corinna Perchtold-Stefan, Eric Rietzschel, Matthijs Baas

**Affiliations:** 1 Department of Psychology, University of Graz, Graz, Austria; 2 Department of Psychology, University of Groningen, Groningen, Netherlands; 3 Department of Psychology, University of Amsterdam, Amsterdam, Netherlands

**Keywords:** malevolent creativity, malevolent innovation, idea evaluation, forecasting, idea selection

## Abstract

Over the last years, malevolent creativity research has delved into creative ideas that intend to harm both on the smaller and larger scale (from creative bullying or deception to terrorism and warfare) in different settings (personal revenge, organizational level, crime). Here, studies have almost exclusively focused on generating/having malevolently creative ideas, either by having people self-report frequency of such ideas, or by measuring their performance on malevolent creativity tests. However, according to stage-based creativity models, (malevolent) idea generation is only one, arguably less consequential aspect of the creative process. Indeed, having harmful creative ideas does not equate assigning them a high value, endorsing them in others, nor implementing them in real-life, which is what ultimately causes societal damage. In this paper, we first summarize the previous research insights on malevolent idea generation, including links to divergent thinking, personality, emotion, and environmental factors. We then offer novel perspectives and avenues for the future of malevolent creativity research by discussing stages beyond idea generation like idea evaluation and forecasting, as well as the endorsement, selection and implementation of harmful creative ideas. Supporting our theoretical arguments, we also include hitherto unpublished research findings from our labs as a basis for discussion. Overall, this paper is intended as a springboard to discuss ways for approximating the malevolent idea journey toward actual idea implementation in malevolent creativity research, i.e., how people transition from generating malevolent creative ideas to executing malevolent creativity actions in real-life (malevolent innovation).

## Malevolent creativity

Ever since Cropley et al.’s (2008) paper “*Malevolent creativity: A functional model of creativity in terrorism and crime*,” research interest in the dark matter has increased exponentially, effectively *shining a light on dark creativity* (Kapoor, 2025). In July 2025, Scopus lists 182 papers on “dark creativity,” summarizing both *negative creativity* (creative ideas that unintentionally or carelessly cause harm; e.g., finding creative ways to skip office work at the expense of colleagues; James et al., 1999) and *malevolent creativity* (creative ideas intentionally used to damage others; e.g., creatively sabotaging a colleague to score a promotion; Perchtold-Stefan et al., 2023a). Moving away from a long-held benevolence bias in creativity research (Cropley and Cropley, 2019; Khessina et al., 2018), this underlines an expansion of investigations into more morally questionable and harmful aspects of creative ideation. While creativity is regarded as a vital skill and the engine of human innovation and flourishing, studies show that creative ideas can be and are effectively used to damage others. This is evident from more extreme examples of novel terrorism, original warfare, or unique crimes (like the 9/11 attacks; Cropley et al., 2008, also see Gill et al., 2013; Hunter et al., 2022; Logan et al., 2023), which combine novelty and uniqueness with effectiveness and usefulness of actions that intend to cause harm.^1^

While extreme examples of malevolent creativity are helpful in outlining the nature of the phenomenon, they may give the impression that harmful ideas are exclusive to extreme individuals (terrorists, criminals, psychopaths, etc.) or settings, and have little relevance in the normative spectrum of human behavior (which may also lead to a serious underestimation of risks). Yet, malevolent creativity is firmly embedded in daily life. Many empirical investigations have explored malevolent ideas and actions in the general population, in the form of creative deception (lying, manipulation), blackmailing, (online) bullying, cybercrime like phishing or catfishing, assault, vandalism, or theft (Cropley and Cropley, 2019; Harris et al., 2013; Harris and Reiter-Palmon, 2015). The implications are grave. Antisocial, aggressive behavior already does extensive damage to individuals and society; however, if this behavior is additionally creative and original, and thus, cannot be reasonably anticipated or prevented, its negative effects are likely amplified (Harris and Reiter-Palmon, 2015; Perchtold-Stefan et al., 2022a). Accordingly, targeted investigations of the causes, consequences, and correlates of malevolent creativity are not abstract endeavors, but constitute highly relevant practical applications of creativity research. Mirroring this idea, scholars seem to fast-track malevolent creativity investigations in recent years. Notwithstanding their importance, the available research has almost exclusively focused on idea generation, i.e., generating or having malevolent creative ideas, measured either by performance on divergent thinking tasks (see Perchtold-Stefan et al., 2021a), or by people’s self-reported frequency of having malevolent ideas (see Hao et al., 2016). Consequently, we seem to have uncovered only a small portion of the phenomenon. Indeed, according to stage-based models of creativity (Mumford et al., 1991; Perry-Smith and Mannucci, 2017; Rietzschel and Ritter, 2018; Wang et al., 2025), idea generation is only one aspect of the creative process, and perhaps, even the least consequential one. Simply having harmful creative ideas (thinking bad; *malevolent ideation*) does not equate assigning them a high value, endorsing them in others, or implementing them in real-life (doing bad; *malevolent innovation*), which ultimately causes damage to society. However, idea evaluation (e.g., Rietzschel et al., 2024), endorsement and implementation (e.g., Hunter et al., 2022) remain understudied in malevolent creativity research.

In this paper, we offer novel perspectives and research avenues for the future of malevolent creativity research^2^ by focusing on *idea forecasting/evaluation* and *idea implementation* as later stages of the malevolent idea journey, and *malevolent creativity perception and endorsement* as (optional) social dynamics of malevolent creativity. Before diving into our arguments, several things should be noted. First, it is not the goal of this paper to detract from previous valuable work on malevolent creativity (including our own); instead, we aim to initiate a fruitful and critical discourse about the future of malevolent creativity research. Second, this paper is neither a systematic nor exhaustive review of the crucial work on malevolent creativity. Third, this paper does not propose a comprehensive framework for assessing the entire malevolent idea journey, but reflects observations made in our labs over the years, and portrays subjective perspectives for where research could go. Moreover, we present unpublished findings based on secondary data from previous publications. This data, while not included in any of our published manuscripts, enabled us to derive empirical research findings to underline our arguments. All corresponding statistical analyses can be found in the Supplementary material.

## Research on malevolent idea generation

### Assessment methods

Along with establishing the concept of malevolent creativity comes the challenge of measurement. Mirroring the reliance on divergent thinking tests in general creativity research,^3^ current attempts to measure malevolent creativity mainly focus on idea generation. Below, we briefly highlight four different measurement approaches to malevolent idea generation, noting some advantages and potential restrictions: the alternate uses test (e.g., Lee and Dow, 2011), real-word divergent thinking tasks without explicit malevolence instructions (e.g., Kapoor and Khan, 2017), self-reported frequency of malevolent ideas (Hao et al., 2016), and malevolent creativity tests with explicit instructions for creative harm (Perchtold-Stefan et al., 2021a). For a more thorough overview on measuring the dark side of creativity, we refer to Kapoor’s (2024) book chapter.

The *alternate uses task* (AUT; Guilford, 1967) asking for original uses of everyday objects has also been adapted to measure malevolent creativity. One approach considers fluency of malevolent ideas (i.e., using a brick as a weapon) as an index of malevolent creativity (Lee and Dow, 2011; later: Dumas and Strickland, 2018). However, as malevolent creative ideas need to be both harmful and original (Harris and Reiter-Palmon, 2015), others have used more refined scoring by incorporating the degree of originality in their AUT malevolent creativity metric (see Baas et al., 2019). Conceptually, this approach aims to measure *unsolicited* malevolent creativity, i.e., malevolent ideas in divergent thinking tasks without any emotional/malevolence prompts. While this unprompted assessment of malevolent creativity is seen as an advantage by some (Dumas and Strickland, 2018), others have questioned its ecological validity: First, there is the low base rate of malevolent ideas in the AUT as a non-social creativity task, capturing little of what may elicit malevolent creativity in real life (Kapoor and Khan, 2018). Second, in fluency-based AUTs, malevolent ideas may simply be a byproduct of versatile creative ideation, making this a measure of negative rather than malevolent creativity (James et al., 1999; Kapoor and Khan, 2018; Perchtold-Stefan et al., 2021a).

To address this criticism, other work used *real-world divergent thinking tasks without malevolence instructions*. Some of these tasks include idea generation to hypothetical open social problems (e.g., *Your close friend’s wedding is coming up. Despite being in town, you do not want to attend it;* see Kapoor et al., 2025, also see Gutworth et al., 2018; Harris et al., 2013; Kapoor and Khan, 2017). Others use laboratory negotiation tasks (Baas et al., 2019), giving participants a wider social canvass for generating creative ideas, but simultaneously hinting at malevolent creativity due to the inconvenient nature of the situations, along with different framing of certain goals that creative ideas should accomplish (also see Kapoor, 2024). While lower frequency of malevolent creativity ideas (compared to explicit malevolence instructions, see below) may complicate measurement, a critical advantage of these tasks is the contextualized modelling of close-to-real-life situations that may elicit unethical or harmful ideation in certain individuals.

Research also assesses malevolent creative ideation by self-report, with the Malevolent Creativity Behavior Scale (MCBS; Hao et al., 2016) being the most utilized measure to date. In this questionnaire, participants report the frequency of malevolent creativity behaviors in terms of hurting others (*“How often do you have ideas about new ways to punish people*”), lying (“…*do you fabricate lies to simplify a problem situation*?,” and playing tricks (“…*do you play tricks on others as revenge*?”). A distinct advantage of this approach is the quick and economic assessment of dark creative ideation, which may explain MCBS’s popularity (see Geng et al., 2024; Fousiani et al., 2025; Hao et al., 2020; Jia et al., 2020; Shi et al., 2023; Xu et al., 2023; Zhang et al., 2024). However, the measure has some restrictions. For one, it does not distinguish between negative (“*lying to solve a problem*”) and malevolent creativity (“*having ideas to punish people*”) or between creative ideation (“*having ideas*”) and creative actions (“*sabotaging others*”; see Kapoor, 2024), complicating interpretation. Moreover, researchers have noted that the items only partly capture originality *and* malevolence, with most focusing on malevolence alone (Reiter-Palmon, 2018; Perchtold-Stefan et al., 2021a; Waldie et al., 2021). While there have long been no other developments on this self-report front, Wang et al. (2025) recently introduced the Malevolent Innovative Behavior Scale (MIBS), which places a stronger focus on novelty and intentional harm in an organizational context and will be discussed later in more detail.

Finally, *real-world divergent thinking tasks with malevolence instructions*, usually called “malevolent creativity tests” (MCTs) have become popular in recent years (see, e.g., Geng et al., 2024; Hao et al., 2020; Perchtold-Stefan et al., 2021a,b; Zhao et al., 2022; Zhang et al., 2024). As a common denominator, malevolent creativity tests typically confront participants with one or several provocative social situations (e.g., “*Your neighbor offers you money for your help in renovating their flat, but after you did the work, they insist that you just imagined the whole thing,”* from Perchtold-Stefan et al., 2021a,b). Then, participants are solicited to generate creative ideas to take revenge on the wrongdoers within a certain time. While idea scoring often varies considerably for different test versions, most are inspired by the work of Harris and Reiter-Palmon (2015), who were the first to explicitly instruct creative revenge taking to solve social problems, along with rating valence and originality of generated ideas. Our lab also drew strong inspiration from this approach as well as psychometric divergent thinking tests, creating a 4-item and later 6-item version of a revenge-based malevolent creativity test that scores fluency, malevolence, and originality, which are then compiled in a total malevolent creativity score (i.e., number of malevolent ideas that are at least moderately original; Perchtold-Stefan et al., 2021a, Perchtold-Stefan et al., 2021b, Perchtold-Stefan et al., 2022a, Perchtold-Stefan et al., 2022b, Perchtold-Stefan et al., 2023a, Perchtold-Stefan et al., 2023b, Perchtold-Stefan et al., 2024). We will note shortcomings of this approach when we discuss our conception of the malevolent idea journey.

In the following, we will highlight empirical research findings with these different measures for malevolent idea generation. For simplicity reasons, we will—where necessary—only distinguish malevolent creativity self-report (by MCBS) and test performance in divergent thinking tasks (e.g., MCTs).

### Correlates of malevolent idea generation

**Figure 1:**
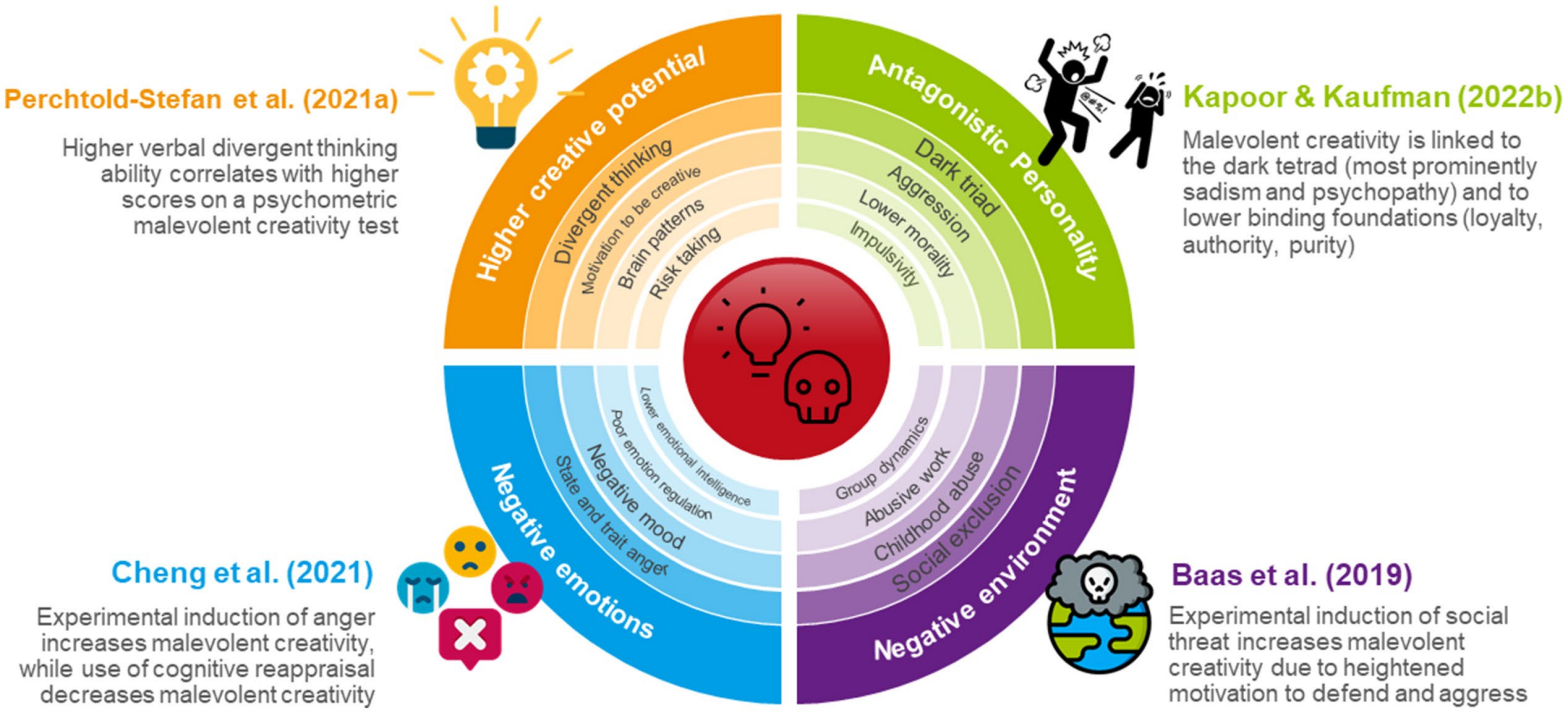
Summary of research findings on malevolent idea generation. Malevolent creativity is linked to higher general creativity (orange), antagonistic personality traits (green), negative emotions and lower socio-emotional skills (blue), and negative environments (purple). Highlighted studies serve as examples of investigations. Larger fonts indicate more general or most researched constructs, while smaller fonts indicate more specific or less researched traits/factors.

Research has attempted to answer the question who is motivated and capable of high malevolent creativity under which circumstances. To help cluster previous insights, we utilize Kapoor and Kaufman’s (2022a) theoretical AMORAL model of dark creativity, which discusses antecedents, (individual) mechanisms, and (environmental) operants of dark creativity, along with realization (e.g., creativity domain), after-effects (e.g., actual valence of actions), and legacy (e.g., achievement of Big-C) of dark creative actions. Figure 1 illustrates respective research findings.

As part of AMORAL’s individual mechanisms (i.e., features, characteristics), being more generally creative—*higher creative abilities and activities*—is associated with greater malevolent creativity (Kapoor and Kaufman, 2022a). With robust correlations between verbal divergent thinking scores and malevolent creativity (Hao et al., 2016; Perchtold-Stefan et al., 2021a; Xu et al., 2023), it follows that malevolent creativity is unlikely without a general propensity for novel thoughts and behaviors. This is further underlined by neurophysiological and brain imaging studies finding quite similar brain activation patterns for general and malevolent divergent thinking (Gao et al., 2025; Perchtold-Stefan et al., 2023a).

Perhaps the most researched individual mechanism of malevolent creativity is personality. A variety of studies have established that individuals high in *antisocial and antagonistic traits* demonstrate higher malevolent creativity, as is evident from links to trait aggression, impulsivity, the dark tetrad, and trait deceptiveness (Lee and Dow, 2011; Hao et al., 2016; Harris and Reiter-Palmon, 2015; Kapoor et al., 2025; Kapoor and Kaufman, 2022b; Perchtold-Stefan et al., 2021a, 2022b, 2024; Szabó et al., 2022). In terms of personal values, Kapoor and Kaufman (2022a,b) also note lower moral foundations (e.g., loyalty, fairness) as a decisive factor for malevolent creativity. Overall, it is easily argued that callous, deceptive, or disinhibited individuals more readily apply their creative capacities for harmful purposes. However, while there is a dark personality core to malevolent creative ideation (see Dow, 2023), we feel that past research may overemphasize the role of antisocial personality in *creative* harm, especially pertaining to extremely rare traits like (clinically relevant) psychopathy or sadism (Cropley, 2025). There is a discrepancy in correlations between dark tetrad traits and malevolent creativity self-report vs. creative performance. MCBS scores show moderate to large correlations with the dark tetrad (Jia et al., 2020; Szabó et al., 2022; Kapoor and Kaufman, 2022b), yet links are significantly reduced or even vanish completely when it comes to other-rated originality in dark creativity tests (Lebuda et al., 2021; Perchtold-Stefan et al., 2024). Accordingly, traits like psychopathy may be linked to malevolent behavior (harming others), but their links to *novelty of harm* are less straightforward.

*Affective states and socio-emotional skills* may also help explain why seemingly “ordinary” people may come up with creative ideas to hurt others, as they likely determine how people construe malevolent creativity test situations, impacting their ideation. While malevolent creativity may certainly be used proactively (i.e., instrumental aggression), reactive incidences in everyday life are more prevalent. Accordingly, researchers noted that malevolent creativity may most likely result from provocative contexts in which people feel wronged, exploited, or threatened, leading to negative affect and a revenge-oriented mindset (Baas et al., 2019; Harris et al., 2013; Harris and Reiter-Palmon, 2015; Perchtold-Stefan et al., 2021a). In support, empirical investigations have linked anger, threat, and negative mood to increased malevolent creativity, both at the trait and the state level (Baas et al., 2019; Cheng et al., 2021; Hao et al., 2020; Perchtold-Stefan et al., 2021a, 2023b). These results fit well with prominent affect models of creativity postulating that activating feelings (whether positive or negative) increase creativity (see overview by Baas, 2019). Anger additionally promotes approach motivation (Harmon-Jones, 2003), which is linked to greater risk-taking and defiance of social norms, likely boosting malevolent ideation in provocative contexts in which “good social conduct” has been broken (Hao et al., 2020; Perchtold-Stefan et al., 2021a). While certain affective dispositions count toward individual mechanisms of dark creativity, so do (absent) socio-emotional skills. Harris et al. (2013) reported higher malevolent creativity in individuals with lower emotional intelligence, suggesting that poor understanding and management of (one’s own and other’s) emotions may lead to malevolent problem-solutions, even in benevolent or neutral situations. Other studies found malevolent creativity linked to poor self-regulation and deficient cognitive reappraisal of anger (Cheng et al., 2021; Perchtold-Stefan et al., 2021b). While much is still to be discovered in terms of other affective components, we may tentatively conclude that negative affect, antisocial personality, and general creative potential serve as predictors of dark creativity, each likely explaining unique variance at the stage of malevolent idea generation (Perchtold-Stefan et al., 2021a).

Lastly, research also considers *environmental and social contexts* that promote malevolent creative ideation (classified under “operants” in Kapoor and Kaufman, 2022a). Gutworth et al. (2018) advised that creative harm is not exclusive to “malevolently creative individuals,” but that anyone may, under certain circumstances, resort to dark creativity. In support of this statement, the authors showed that malevolent situational cues increased malevolent creativity independently from cognitive abilities and personality traits (Gutworth et al., 2018). Later studies further underscored the power of social contexts for malevolent creativity. Baas et al. (2019) found that participants threatened with substantial losses in prisoners’ dilemma games (high social threat) generated more malevolently creative tactics in a subsequent negotiation task. Similarly, Perchtold-Stefan et al. (2022a) found that participants randomly assigned to an experimental social exclusion (vs. inclusion) condition showed higher malevolent creativity in a subsequent revenge task targeting unrelated others. Recently, Fousiani et al. (2025) manipulated fear of power loss in leaders dealing with insubordinate employees. Leaders assigned to the high fear of power loss condition and low in responsibility experienced greater anger at employee insubordination, which increased harmfulness of creative ideas generated to solve the problem. To explain such effects, researchers have suggested that threatening and provocative contexts may trigger a reduced motivation for self-control, attempts to regain power, stronger aggressive self-defense, and higher persistence in resolving threating situations as context-specific drivers of malevolent creativity (Baas et al., 2019; Perchtold-Stefan et al., 2022a). While these studies addressed current social context in malevolent creative thinking, other investigations target the role of early life environments in shaping malevolent creativity. Here, research points to a link between negative childhood experiences (like abuse, neglect, or trauma), and higher malevolent creative ideation in adulthood. This association is interpreted as childhood adversity fostering the development of hostile attribution styles, moral disengagement, and antisocial traits (see Jia et al., 2020; Li et al., 2022). Altogether, while environments naturally interact with individual mechanisms of cognition, personality and emotions in explaining malevolent idea generation, we consider the increased research focus on contextual daily-life operants a fruitful approach to understanding everyday “little-c” harmful creative ideation (Gutworth et al., 2018; Kapoor and Kaufman, 2022a; Perchtold-Stefan et al., 2022a).

### What idea generation research does not (quite) tell us about malevolent creativity

Notwithstanding the tremendous ground covered by research in recent years, understanding malevolent idea generation is only the beginning of the malevolent idea journey. In the organizational creativity literature, the ‘idea journey’ (Perry-Smith and Mannucci, 2017) refers to the entire idea trajectory from conception to implementation. Research on the “receiving side of creativity” (Zhou et al., 2019) focuses on identifying factors and processes that could help creators move through the idea journey more effectively—e.g., finding the social and informational support to help elaborate their ideas, or gaining access to resources (through effective championing) to work on implementation. When it comes to malevolent ideas, in contrast, one would hope to *prevent* completion of this idea journey—but in both cases, a deeper understanding of the relevant facilitators and obstacles is important.

Previous research has reported positive correlations between malevolent creativity performance and self-reported behavior in questionnaires (Hao et al., 2016; Kapoor and Kaufman, 2022b; Perchtold-Stefan et al., 2021a), which is usually taken as evidence that people scoring higher on a malevolent creativity test also tend to act more malevolently creative in daily life. However, there are several caveats to this. First, correlations between malevolent creativity tests and self-reports are rather small (*r*’s from 0.14 to 0.36, Hao et al., 2020; Perchtold-Stefan et al., 2021a; Shi et al., 2023; Zhao et al., 2022), suggesting that the link is not very strong. Second, self-ratings of malevolent idea implementation (more generally in the MCBS) or in specific work contexts in the MIBS (Wang et al., 2025) only access a subjective part of idea implementation that is perhaps, a bit too far removed from what individuals actually do in real-life. As we know from general creativity research, self-perceived creativity and actual creative achievements are not highly correlated (see Haase et al., 2018; Said-Metwaly et al., 2022). Third, performance on malevolent creativity tests may not reflect actual behavior. In hypothetical test scenarios soliciting original revenge ideas, people may present higher malevolent creativity for many reasons unrelated to their real-life behavior. For one, individuals may apply their general creative capacities to malevolent creativity tasks in compliance with test instructions, i.e., to please the experimenters (see Dumas and Strickland, 2018). While high task engagement may not necessarily increase malevolence of ideas, it may certainly affect the sheer production of ideas, and thereby idea originality (cf. De Dreu et al., 2024; Rietzschel et al., 2007). Another reason for higher scores on malevolent creativity tests may be acquired passive knowledge on creative aggression from others’ behaviors in real-life or media contexts. Perchtold-Stefan et al. (2024) found that police officers working in a maximum-security prison significantly outperformed prison inmates in malevolent idea generation, which could be interpreted as a richer passive repertoire of ideas accumulated by observing criminals’ behaviors. Lastly, malevolent idea generation may be a fun thought experiment, as it may be interesting to entertain taboo ideas in a safe setting without any consequences.

### Other aspects of the malevolent idea journey

The problem of equating malevolent idea generation with implementation is further underlined by studies on violent behavior, showing that links between aggressive ideation and aggressive outcomes are tenuous. In one of the rare studies differentiating malevolent ideation and innovation, Hunter et al. (2022) note that violent thoughts (about hurting others; intrusive thoughts of stressed parents regarding their infants) are rarely linked to actual acts of violence. Similarly, while revenge fantasies are common in the general population, the frequency of actual retaliatory behavior is much lower (see Crombag et al., 2003), which highlights the need to look beyond malevolent idea generation and capture other aspects of the malevolent idea journey. Finally, we know from general creative idea journey models that environmental factors and individual differences that influence idea generation may not influence other stages in the idea journey, including the implementation of an idea (Perry-Smith and Mannucci, 2017; West, 2002). Importantly, our stance in this argument is that the available tools for assessing malevolent idea generation are solid (Perchtold-Stefan et al., 2022a; Wang et al., 2025) and have delivered a respectable profile on this stage. Now, the goal is to supplement them with more fine-tuned measures that also capture idea perception, idea evaluation/forecasting, and idea implementation, i.e., the entire process through which malevolent creative ideas turn into actual products and behaviors, which is called *malevolent innovation* (Hunter et al., 2022; Nguyen et al., 2024). Research with these measures will then enable a more comprehensive picture of the individual differences and environmental factors that impact the different stages of the malevolent idea journey.

### Problem construction in malevolent creativity

Contrary to popular belief, the creative process does not start with idea generation, but with problem construction (also called problem finding) as real-life problems that require creative solutions are usually complex, ill-defined, and may include conflicting goals and interests of involved parties (see Reiter-Palmon et al., 2024). Accordingly, at the beginning of creative problem-solving, before idea generation, the problem solver is tasked with identifying a problem, as well as structuring and defining often ambiguous, ill-defined parameters, including the task goal (e.g., Mumford et al., 1996; Reiter-Palmon and Robinson, 2009). Research shows that problem construction is positively linked to creativity: people better at defining problems score higher on creativity tests (mean *r = 0.*29), along with reporting more creative personalities and products (*r’s* = 0.23–0.25; see a meta-analyses by Abdulla et al. 2020, 2023) This link seems quite substantial, given that relationships between divergent thinking in laboratory tasks and creative achievement in real-life are similar in size (Kim, 2008).

Creativity research is only catching up to this early creative problem-solving stage, and Reiter-Palmon et al. (2024) discuss that the mechanism through which individual problem construction improves creative outcomes is largely unexplored. However, if problem construction influences subsequent creative ideation (Abdulla et al., 2023), this may also be of critical interest to malevolent creativity research. Though empirical research is pending, Harris et al. (2014) speculate that individuals with aggressive tendencies or aggressive expertise (e.g., influenced by personal negative life events) may construct problems in a more destructive way that justifies harmful creative solutions, making malevolent creativity seem more appropriate. Harris and Reiter-Palmon (2015) found that individuals high in implicit aggression, while generating more malevolently creative ideas in response to aggressive problems, did not show higher malevolent creativity in response to prosocial problems, presumably because the contextual cues did not elicit negative problem construction. Consequently, Harris et al. (2014) argue that malevolent creativity is not only based on having harmful intent, but also a matter of how certain individuals, based on their personality and past experiences, define problems in the first place (e.g., *what exactly is the problem with my neighbor not paying me for my work in their flat?*).

Although research on malevolent problem construction tasks is currently missing, insights can be inferred from participants’ (unsolicited) reflective answers on idea generation tests. For instance, Kapoor et al. (2025) show that there is a virtue caveat in divergent thinking tasks with unethical instructions, underlining that some participants explicitly refuse negative responses as a task goal (e.g., “*I will not think of anything unethical*”; also see Perchtold-Stefan et al., 2024). In addition, in the revenge tasks soliciting malevolent creativity in our lab (Perchtold-Stefan et al., 2021a,b), some participants described problem-discovery to address the unfair neighbor situation (e.g., *I will have an extensive talk with them and ask what’s wrong*), engaged in cognitive reappraisal of the problem^4^ (e.g., *This is not that bad, I can just put it down as a good deed*), or reconstructed the problem altogether (e.g., *“The problem is not the lack of money, but that I did not get this in writing in the first place”*). While these answers were initially surprising to us (as task instructions explicitly asked for harmful creative ideas), we now feel that different problem construction in malevolent creativity could be a very fruitful area of investigation, especially when participants are tasked with creative solutions to more ambiguously framed situations, or instructions emphasize creative but not explicitly malevolent solutions (see Fousiani et al., 2025). The potential for creativity often lies in the ambiguity of situations (with different people seeing different opportunities or risks). However, ambiguity may also engender malevolent creativity in the sense that if (organizational) norms and procedures are not clear, people show more deviant behavior. Future research may use problem construction tasks (e.g., Mumford et al., 1996; Reiter-Palmon et al., 2024) and uncover both individual differences (e.g., prosocial tendencies, trait aggression) and environmental factors (e.g., task instructions, problem information) as predictors of malevolent creativity.

### Idea forecasting and evaluation in malevolent creativity

On the route to malevolent innovation, many things can go wrong along the way (see Rietzschel et al., 2006; Rietzschel and Ritter, 2018 making this argument for general creativity and innovation). In their systematic review on *creativity evaluation and selection*, Rietzschel et al. (2024) note that both these processes form a critical, yet largely neglected bottleneck in the innovation process, with many people generally performing suboptimally in evaluating and poorly in selecting their own creative ideas (see Rietzschel and Ritter, 2018). While in the case of malevolent creativity, we would hope for this outcome, i.e., creators failing to recognize high quality of creative harm (e.g., *a crime writer not realizing that a creative scam they invented for their protagonist could also be highly useful in real life*), there is barely any empirical research on how people may forecast consequences and challenges of their generated malevolent ideas, and how they attribute value to them. Notwithstanding the knowledge gained from idea generation, in the understanding of malevolent innovation as a whole, there seems to be a conceptual disconnect if we ask participants to produce highly creative ideas for taking revenge (Perchtold-Stefan et al., 2021a), but do not follow up with what they do with these ideas afterwards. Accordingly, we would like to draw attention to idea evaluation and forecasting as promising stages of the malevolent idea journey.

Most influential process models of creativity include idea evaluation as a precursor to idea implementation, proposing that idea generation is followed by forecasting possible implications, assessing the viability of the idea, and then either deciding to further develop or implement the idea or dropping it entirely (see, e.g., Mumford et al., 1991; Mumford et al., 2002). Accordingly, idea evaluation means assigning a certain value to an idea, either based on one or multiple criteria (e.g., how creative, how useful, how risky) and is still quite far removed from (malevolent) idea implementation, as it requires no real decision, but is simply a judgment of certain idea qualities (Rietzschel et al., 2024). Still, also for malevolent creativity, this is likely a crucial step for deciding which ideas will be developed further, and which ideas will be discarded. Highlighting the significance of this stage, work by Mueller et al. (2012, 2018) in organizational contexts showed that “just a few tweaks” to social roles, mindsets, and contexts of decision making significantly affected evaluation of creative ideas. For instance, this work showed that environmental uncertainty lowered evaluations of creative ideas. While we cannot fully emulate real-life decision making in laboratory tasks, hypothetical malevolent creativity tests can manipulate ambiguity, uncertainty, and even social approval along the malevolent idea journey. This way, asking participants how original, useful, or effective they consider each of their generated ideas may provide vital insights on the malevolent ideator’s mind that research has neglected so far.

Another interesting step between idea generation and implementation is creative forecasting. Here, participants are instructed to predict the actual outcome of idea implementation (e.g., the perceived success, costs, etc.; Rietzschel et al., 2024). While some creativity models place forecasting at the beginning of the idea evaluation stage (e.g., Mumford et al., 2002), others consider it closer to idea selection (Rietzschel et al., 2024), as it draws more on people’s expectations of how their ideas will be perceived and function in real-life, and less on their opinions about their ideas. Either way, research suggests that forecasting may critically affect creator’s decision processes all the way to the implementation of their ideas, as more extensive forecasting (of both positive and negative idea outcomes) has been linked to higher quality, originality, and effectiveness (elegance) of finalized ideas (Byrne et al., 2010; McIntosh et al., 2021). With forecasting, the interesting thing is that people may believe an idea to be highly original, but still failing to meet an important goal in practice (due to lack of resources, risk of social backlash, etc.). While idea forecasting is only gaining ground in creativity research (see Berg, 2016, 2019; McIntosh et al., 2021), it may hold special value for malevolent creativity, as people conduct a cost–benefit analysis and have to contrast their own personal gains with projected real-world consequences of their harmful ideas–also relevant for how willing people are to share their ideas with others. Overall, asking participants how successful they think each of their ideas would be in reality (regarding costs and benefits) seems very informative regarding their real-world perceptions and the contextual connotations of malevolent creativity. Taking the unfair neighbor item for example (Perchtold-Stefan et al., 2021a,b), it is likely that participants may evaluate their most harmful idea as very effective for taking revenge (e.g., *sending them a gift from “their mother” that incinerates itself*, *burning the flat down*), but would forecast the highest success for a milder idea (e.g., *spreading rumors that the neighbor has an online career on adult websites*) based on a risk–reward analysis. We also note with interest that, similar to general creativity, forecasting in malevolent creativity may be prone to all kinds of errors (e.g., overestimating the impact of well-made deep-fake video of a person, as people have become more aware). Ultimately, the inclusion of forecasting questionnaires such as those by McIntosh et al. (2021) are needed to reveal these insights.

Scrutinizing creative forecasting may also deliver new insights into reported associations of dark personality traits with malevolent creativity (e.g., Kapoor and Kaufman, 2022b). For instance, we speculate that individuals higher in narcissism may generally over-evaluate the original deception of their generated ideas, forecasting success for malevolent ideas that are, in fact, easily spotted by others, thus failing at the implementation stage. By comparison, individuals higher in (secondary) psychopathy, especially when provoked, may not value creativity, but instead, harmfulness of ideas to do maximum damage, forecasting higher success for uncreative but highly malevolent ideas, making them high in aggression, but low in malevolent innovation. Finally, Machiavellianism (higher in self-control and calculated exploitation of others) may not necessarily be linked to the highest creativity in fluency-based malevolent idea generation, yet may enable a more realistic assessment of single ideas high in creative harm, which are more strategically selected for implementation (for malevolent creativity and the dark tetrad, see Dow, 2023).

As noted in the beginning of this paper, we aim to underline some of our theoretical arguments on the malevolent idea journey with findings derived from unpublished empirical data from our labs. To better distinguish between theoretical claims and empirical findings, we subsequently highlight empirical sections with the subheading “From theory to empirical data.” Importantly, it should be noted that our mentioned studies only serve as preliminary, but certainly not conclusive empirical support for our ideas. For study 1, see Box 1.

BOX 1 **From theory to empirical data–study 1^5^**

Forecasting may also be used to better understand circumstances in which people may actually consider implementing their generated ideas on hypothetical malevolent creativity tests. We asked participants (*n* = 160) to first generate creative ideas to take revenge on wrongdoers, and then forecast which individual mechanisms (e.g., hurt pride, anger) and social contexts (e.g., distance to wrongdoer) would cause them to implement their ideas in real-life (10 mechanisms/contexts in total; 1 = would likely not implement, to 5 = would very likely implement). Participants forecasted the highest likelihood for idea implementation if friends or family members were hurt by the wrongdoer (*M* = 4.11), followed by the wrongdoer not showing remorse for their behavior (*M* = 3.71), and wanting to restore justice (*M* = 3.42). Conversely, the situation threatening one’s world view (*M* = 2.43), distance to the wrongdoer (*M* = 2.54) and other witnesses (*M* = 2.61) received the lowest implementation likelihood ratings. However, participants with a higher capacity for malevolent idea generation on the MCT more likely forecasted the implementation of their ideas in situations of larger distance to the wrongdoer (*r = 0.*21, *p = 0*.007), lack of negative consequences of doing creative harm (*r = 0.*17, *p = 0*.029) and if they felt the need to restore justice (*r = 0.*18, *p = 0*.020). Conversely, people reporting higher trait aggression and narcissism more likely forecasted implementation of ideas in situations of high anger (aggression: *r = 0.*24, *p = 0*.003; narcissism: *r = 0.*15, *p = 0*.056), with witnesses (aggression: *r = 0.*23, *p = 0*.004; narcissism: *r = 0.*18, *p = 0*.023), and if their pride was hurt (aggression: *r = 0.*22, *p = 0*.006; narcissism: *r = 0.*025, *p = 0*.001). See Supplementary part A. While this only serves a preliminary indication that forecasting could be useful for understanding malevolent innovation, it hints at interesting differences in how certain individuals may transition from malevolent ideation to innovation. Tentatively, while people with higher creative capacities may transition to implementing malevolent ideas under low stakes and punitive motives, people high in trait aggression and narcissism may transition to idea implementation under high emotional load and threat to status.

### Idea perception and endorsement in malevolent creativity

People’s own encounters with malevolent creativity in daily life are often online. Tales of clever deceit, unique aggression, and malicious pranks are all over the internet: a woman going viral on TikTok for hiding stinky shrimp in her cheating ex-boyfriends’ curtain rods, or people dressing up as killer clowns and stalking others, earning millions of views, with some calling these actions “novel,” and “worthy of imitation.” In 2024, an “animalistic” attempted fraud turned media hype, as bears damaging cars in Los Angeles turned out to be humans in bear costumes trying to cash an insurance check. Why are these examples important for understanding the malevolent idea journey, and by implication, the dangers of malevolent innovation? Simply put, the benevolence bias may paradoxically also hold for malevolent creativity. Creative ideas and personalities may elicit awe and admiration, and may evoke positive associations per default (Baas et al., 2015; Sternberg, 1985), since they are innovative, entertaining, and amusing. Cropley et al. (2014) reported that laypeople perceived both ambiguous and illegal/violent actions as more creative than illegal/nonviolent actions, showing that individuals can appreciate creativity in harmfulness. Further, people creatively bending rather than outright violating rules are seen as relatively high in both prestige and dominance, making them more attractive as leaders than (uncreative) rule-breakers (Homan et al., 2024). Similarly, Wiltermuth et al. (2017) showed that creative unethical behaviors were judged more leniently by others than uncreative behaviors, presumably because people attribute higher competence to creativity, which more positively colors moral judgments. Accordingly, aspects of power, competence, and entertainment value of ideas may overshadow their potential harm (for empirical studies, see McGraw et al., 2014; Sawada and Nomura, 2020), which is why malevolent creative ideas and actions may be rated as less serious, damaging, and less worthy of punishment. This not only has grave implications for the victims, but may also incentivize more malevolent creativity in the future due to fewer sanctions. For these reasons, we argue that perception and endorsement of malevolent creativity may be especially relevant at two stages of the malevolent idea journey: after idea generation (endorsement of malevolent ideation) and after idea implementation (endorsement of malevolent innovation). While the examples above (shrimp, killer clowns, bears) illustrate endorsement of malevolent innovation, there are also countless instances in which malevolent ideas are first communicated to others, and then implemented depending on social feedback, e.g., online creators announcing creative pranks by stating, “If this idea video gets 10,000 likes, I’ll do it.”

In their paper on the MIBS as a novel self-report measure for malevolent innovation in the workplace, Wang et al. (2025) emphasize the importance of malevolent creativity endorsement–which they call *championing*–in the malevolent idea journey. In their framework, idea championing is a decisive factor for malevolent ideas transitioning to malevolent actions (e.g., *a company seeking support/approval from stakeholders to remove a competitor from the market*). Strictly speaking, championing can refer to two different behaviors from different persons: soliciting support by the creator and giving support to the creator (Wang et al., 2025). In our example, the company liaison can champion their malevolent idea to get stakeholders behind them, and stakeholders can champion the idea by signaling their support. In line with this, several takes on the creative idea journey in organizational contexts argue for the relevance of idea championing as the active promotion of one’s creative ideas (on part of the creator) and the resulting perception/endorsement of others, giving the idea legitimacy and facilitating implementation (Caniëls et al., 2014; Mueller et al., 2018; Perry-Smith and Mannucci, 2017). Transferred to malevolent creativity, we thus argue that the way a harmful idea is communicated, received, and socially accepted/supported (or not) should be more strongly considered in explaining how people move from malevolent ideation to malevolent action. Importantly, we consider idea endorsement/championing a highly interesting, yet optional stage in the malevolent idea journey. While idea implementation always requires idea generation first, not all creative processes operate in a social setting. As stated by Wang et al. (2025), an individual may move from ideation to implementation without requiring endorsement/championing (e.g., *changing a co-worker’s documents into an unreadable font*), whereas at the group level or for ideas requiring substantial resources, championing may be necessary. Still, in line with the saying that “evil will triumph when good men do nothing,” implementation of a malevolent idea may not so much depend on others explicitly supporting it, but rather on the lack of pushback. Similarly, to what is seen for bullying, perpetrators may be able to persist in their malevolent behaviors because nobody explicitly tells them to stop, or because there are no systems in place to force them to do so.

In their AMORAL model, Kapoor and Kaufman (2022a) note material (e.g., access to facilities) and social assets (e.g., leveraging social circles) as environmental operants of dark creativity, which may tap into the endorsement stage of malevolent creativity and “make or break” an idea. Hunter et al. (2022) surmise that beliefs in the righteousness of malevolently creative acts often emanate from social influence, with social connections and support propelling malevolent ideas toward implementation. In sum, even if idea endorsement is not part of every malevolent idea journey, it may still explain considerable variance in why some individuals turn their malevolent creative into action, and why other attempts fail after the ideation stage (Hunter et al., 2022; Mueller et al., 2018; Wang et al., 2025). From a temporal perspective, it is possible that malevolent idea endorsement may either come into play in between idea generation and evaluation (e.g., *a spontaneously uttered idea for revenge receives immediate support by a friend*), between evaluation and implementation (e.g., *a carefully crafted plan of sabotaging a supervisor falls apart due to colleagues’ objections*), or both. For study 2, see Box 2.

BOX 2 ***From theory to empirical data–study 2*^6^.**

We have started to investigate a central premise of our arguments–that people may have a benevolence bias: due to their novel and entertaining nature, malevolent creative ideas may be regarded as less harmful and receive more endorsement compared to ordinary aggression. In a pilot study, we created a pool of 92 items reflecting 46 pairs of aggressive ideas/behaviors, with one describing a generic (noncreative) aggressive idea/behavior (e.g., *stealing a roommate’s diary and use their secrets to blackmail them*) and one describing a creative version of the same idea/behavior (e.g., *using a therapy chatbot that acts helpful, but extracts the roommates’ personal secrets for blackmailing*). For the pilot run, *n* = 21 participants were presented with these ideas in randomized order and rated them for various criteria. As a successful manipulation check, items including creative aggression were rated as significantly more creative than items including ordinary aggression (*p* < 0.001, *d =* −2.83). While we observed no significant differences for ratings of physical (*p = 0*.743, *d =* 0.07) and mental harm (*p = 0*.746, *d =* 0.08), participants rated the short-term consequences of creative aggression as less severe (*p = 0*.021, *d =* 0.55), in addition to perceiving creative aggression as more surprising (*p* < 0.001, *d =* −1.82) and humorous (*p* < 0.001, *d =* −1.79). Most importantly to us, creative aggression received higher ratings of endorsement (*p = 0*.019, *d =* −0.56) as well as likelihood of liking (*p* < 0.001, *d =* −1.21) and sharing creative aggression (*p* < 0.001, *d =* −1.35) on social media. See Supplementary part B.

While the findings of our study 2 are based on a small pilot sample—a pre-registered large-scale investigation is currently in progress–they preliminarily confirm that malevolent creativity may receive stronger endorsement/championing than noncreative aggression, corroborating the need for further investigations at this stage. Focusing on individual mechanisms (Kapoor and Kaufman, 2022a), it may be interesting to see if idea championing for ordinary vs. creative aggression is moderated by specific traits, values, or emotional characteristics, and thus depends on people’s belief system and moral-ethical framework. For this reason, our pre-registration also includes measurement of dark personality, moral disengagement, or negative affect. However, there are also interesting studies to be done from the perspective of explicitly soliciting support for malevolent ideas on part of the creator, as some people may be better solicitors than others. Goncalo et al. (2010) found that while narcissism did not predict individual creative performance, it did predict how others perceived the creativity of ideas, with ideas of similar quality rated as significantly more creative when they were pitched by a narcissist. While links between narcissism and malevolent idea generation are still vague, the results by Goncalo et al. (2010) suggest that such individuals may be able to present harmful creative ideas in ways that appear favorable to others, which is especially relevant for group aspects of malevolent innovation (see Hunter et al., 2022).

### Idea selection and intended implementation in malevolent creativity

The next step closer to *malevolent innovation* (actual implementation of ideas) that follows idea generation, evaluation, and potentially, endorsement, is idea selection, which entails definitive decision making regarding which generated ideas to choose and which to discard (Rietzschel et al., 2010, 2014). While idea selection does not equal idea implementation, it is arguably closest to it, as participants need to converge multiple criteria (e.g., originality, usefulness, effort, consequence, etc.) into a single yes or no decision, which also implies a commitment to one or more selected ideas. Mueller et al. (2018) underline the precariousness of this stage by framing the decision maker’s dilemma of, e.g., choosing between creativity and social approval of ideas–often to the detriment of creativity. While for malevolent creativity, we would like for such dilemmas to halt the malevolent idea journey altogether, we barely know which of their generated malevolent ideas participants’ ultimately find attractive enough to select, and we know even less about why. Notably, this is also where data from our lab already shows a disconnect between idea generation and intended implementation. For study 3, see Box 3.

BOX 3 **From theory to empirical data–study 3^7^:**

In two previous studies, after having generated creative ideas for revenge, we had asked participants to indicate if they would implement these ideas in real-life (yes or no; idea selection). In study part a (Perchtold-Stefan et al., 2021a), of *n* = 107 participants, only 18.7% indicated “yes,” while 81.3% indicated “no.” Interestingly, participants indicating “no” showed higher malevolent creativity test performance than those indicating “yes” [*t*(105) = 3.04, *p = 0*.005], meaning that a higher theorical capacity for harmful idea generation was negatively linked to intended idea implementation. In study part b (Perchtold-Stefan et al., 2021b), this pattern was repeated, with a merely 7.1% out of 79 participants indicating “yes” for idea implementation, suggesting a similar disconnect between idea generation and intended implementation. See Supplementary part C. Naturally, a binary measure of implementation intention is very limited in its informational value; using more granular scales or probability-based ratings would be a valuable next step.

Nguyen et al. (2024) took a more elaborate approach to linking malevolent idea generation with implementation intentions. The authors had participants generate ideas to tarnish the image of a rival university and then indicate desire to implement their plans, using a scale from 1 (would not implement) to 5 (to a large extent). In three studies, malevolent creativity on the task and the desire to implement the generated ideas either showed no or small positive correlations (r’s from −0.10 to 0.29), with the authors concluding that the link between malevolent ideation and intended implementation was nuanced (Nguyen et al., 2024). This shows that a large portion of generated harmful ideas, even in hypothetical tasks may not even be selected for hypothetical implementation, and are thus, far removed from actual malevolent innovation (see next paragraph). Still, research on this is rare, and there are likely many relevant factors that need to be measured when trying to identify key bottlenecks in the transition from idea generation to implementation intentions (e.g., risk preferences, self-control, moral disengagement). There may also be some methodological challenges next to the obvious reliance on participants’ self-report. As Rietzschel et al. (2024) point out, idea evaluation and selection can become highly taxing if embedded in divergent thinking tasks, especially if participants generate many ideas beforehand (also see Rietzschel et al., 2010). Thus, instead of participants making selection and implementation decisions for each of their (manifold) generated ideas, it could be a more convenient option to have them choose from a predefined set of (creative) ideas for harm, to see how different personalities and different features of ideas affect reported implementation intentions. In this vein, Kapoor (2015) had participants choose between three options (neutral, positive-creative, negative-creative) for responding to social problems (e.g., *having borrowed a book from a friend and losing it*), based on what they would most likely do in real-life (idea implementation). While participants selected negative-creative solutions the least, those higher in psychopathy more likely chose negative creativity, while higher narcissism was linked to choosing positive creative solutions; however, as Kapoor (2015) suggests, likely due to feigning both goodness and creativity. It may be an interesting avenue of future research to bridge intrapersonal and interpersonal malevolent idea selection, considering aspects like creative ownership, influence of others’ examples, or psychological distance to ideas (see studies by de Chantal et al., 2023; Norton et al., 2012). Overall, for malevolent creativity tests, we believe that more sophisticated measures of post-generation idea selection and choices on implementation intentions may approximate which ideas people may actually implement in real-life, given that they have also engaged in substantial evaluation and forecasting of the usability, success, and consequences of each idea. In this respect, future path analyses from idea generation to evaluation, (potential) social endorsement, and finally, idea selection, should consider both, characteristics of the creator and of the ideas themselves (originality, malevolence, feasibility, etc.).

### Malevolent innovation

Idea implementation or innovation refers to the intentional execution of creative ideas (Amabile, 1988; Anderson et al., 2014), in our case, actual real-word malevolent creative actions. In their 2022 paper on the link between *malevolent creativity and malevolent innovation*, Hunter et al. draw attention to the fact that 70%–90% of generated creative ideas are never realized (also see Hunter and Cushenbery, 2015; Klein and Knight, 2005; Rietzschel et al., 2024). Together with the weak link between violent fantasies and violent outcomes, this is eminently relevant for our understanding of malevolent creativity (Hunter et al., 2022; Nguyen et al., 2024). In their AMORAL model, Kapoor and Kaufman (2022a) highlight the many layered malevolent creativity journey on a continuum from original and malevolent thoughts (e.g., pure revenge fantasies) over communicating ideas to others (e.g., talking about it with a friend) to spreading creative misinformation online (physically distant behavior), and finally, engaging in original physical violence in real-life (e.g., a creative murder). Having arrived at the presumed end of the malevolent idea journey with the knowledge that malevolent idea generation does not equal implementation, the pressing question is: how do we measure actual malevolent innovation (and the trajectory thereof)? An obvious possibility is individuals self-reporting their harmful creative actions or products, or even self-reporting general deviant behavior, to test for correlation with malevolent idea generation on hypothetical tasks (see Hao et al., 2016; Perchtold-Stefan et al., 2021a). For study 4, see Box 4.

BOX 4 **From theory to empirical data–study 4^8^:**

In a previous attempt to relate malevolent creativity test performance to actual behavior in real-life, we tested relationships between malevolent creativity potential and self-reported misconduct during high school and during the last month (e.g., crime, drug abuse, driving misconduct, etc.). In our study, we did not observe significant correlations between total malevolent creativity and misconduct (high school: *r* = 0.11, *p* = 0.261; current: *r = 0.*10, *p = 0*.297). Looking at misconduct subscales, general criminality (e.g., stealing, selling drugs; *r = 0.*24, *p = 0*.014) and high school misdemeanor (*r = 0.*20, *p = 0*.044) positively correlated with higher malevolence (i.e., harmfulness) of generated ideas. However, as Hunter et al. (2022) critically point out, neither deviance (e.g., generating shocking art) nor criminality (e.g., cashing a fraudulent check) are malevolent creativity if they lack elements of novelty or harmful intent (also see Perchtold-Stefan et al., 2024). Still, in our study, general criminality showed a trend level positive correlation with total malevolent creativity of revenge ideas (*r = 0.*18, *p = 0*.072), suggesting that malevolent creativity tests may capture a certain albeit very small aspect of real-life behavior after all (see Supplementary part D).

Nonetheless, while self-reports of (past) malevolent innovation are useful for assessing what people *believe they (will) do*, they may not quite tap into what people actually *can and will do* (see Wang et al., 2025). As an alternative approach, Hunter and colleagues have conducted in-depth investigations into malevolent innovation in terrorist and extremist organizations, which utilize retrospective analysis of person and process factors leading to creative terrorist acts (see Gill et al., 2013; Hunter et al., 2022). Among others, these works list mental fixation, authority deference (compliance with violence norms), competition, or perceived necessity (e.g., self-preservation) as factors propelling ideas from generation and evaluation to willingness for malevolent implementation, with networks and expertise fueling implementation ability (see Hunter et al., 2022). Some of these aspects (e.g., closed networks, resources, strong management) can also be found in earlier literature on innovation in organizational contexts (Klein and Knight, 2005; Perry-Smith and Mannucci, 2017). While this research targets (malevolent) innovation more at the (extreme) group level, it may be relevant for individual malevolent creativity in the general population as well. However, the question remains how we can test this. As a fundamental issue, in hypothetical tasks we can never fully assess if people actually follow through with their generated ideas and there are obvious ethical constraints to promoting malevolent innovation in experimental settings, even with extensive debriefings afterwards (see Nguyen et al., 2024). Accordingly, we need to find better alternatives that allow the study of malevolent idea implementation apart from self-report, but also without incentivizing real-life harm. Some prospects may lie in competitive gaming and VR simulations. In sum, we should be diligently working on expanding malevolent creativity research by following-up malevolent idea generation with measures of idea evaluation/forecasting, idea selection, and (intended) implementation.

## Conclusion

We propose a reorientation in malevolent creativity research: following the entire malevolent idea journey. This entails looking beyond harmful creative ideation and honing in on other stages that lead to harmful innovation. While generating malevolent ideas remains a fundamental stage of malevolent creativity, we contend that it is merely a springboard for a complex cascade of collateral cognitive, motivational, and emotional processes, which may play out at stages of problem construction, idea evaluation and forecasting, idea endorsement, and ultimately, idea implementation–all of which warrant deeper theoretical and empirical engagement. What we hope to have shown in this paper is that the truly consequential moments of malevolent creativity often do not lie in the generation of an idea, but in what happens next: how individuals perceive its value, how others endorse or discourage it, and how (or whether) it materializes into real-world actions. As argued by research, 70–90% of creative ideas never turn into actions, which shows how nuanced, complex, and multilayered the malevolent innovation process can be. In our opinion, given the extent to which a principally neutral asset like creativity can be realigned from positive to malevolent purposes, it is paramount to understand how darkness gets injected into the malevolent idea journey–and why some ideas are propelled to full distance of malevolent innovation while others lose momentum along the way. This is not only a matter of contemporary academic curiosity but of eminent societal relevance as well.

## Data Availability

The raw data supporting the conclusions of this article will be made available by the authors, without undue reservation.

## Footnotes

^1^While usefulness in malevolent creativity is occasionally debated, research largely takes the stance that benefits of creative actions are highly subjective (Cropley et al., 2008; Cropley and Cropley, 2019) and that usefulness of ideas may vary across social contexts (Plucker et al., 2004). Accordingly, malevolent ideas may be highly useful for people seeking to inflict harm, which is why terror like the 9/11 attacks–morals and emotions aside–reflects high malevolent creativity.

^2^These suggestions may also apply to negative creativity or dark creativity research more generally. However, as methodological conception and measurement of malevolent creativity is more defined, and in line with our own research interests, we subsequently focus on malevolent creativity.

^3^Although there is nothing “general” about creativity, this publication uses the term to distinguish creative ideation without negative/malevolent intentions or applications.

^4^Malevolent creativity appears tied to appraisal: without perceiving a situation as threatening or unfair, people may generate creative but not malevolent ideas, suggesting a dual aspect at this stage: While specific problem construction may facilitate the generation of creative ideas, certain appraisals may push people into a malevolent direction.

^6^This data is not part of any other publications.

^5^The data on the malevolent idea generation task used in this study is also part of another manuscript currently under review. Findings with forecasted idea implementation and personality are exclusively reported in the present manuscript.

^7^This data is not part of any other publications.

^8^The data on the malevolent idea generation task used in this study has previously been published in Perchtold-Stefan et al. (2021a). Links to misconduct are exclusively reported in the present manuscript.
