# Supplementary material for: Generating, evaluating, endorsing, and implementing malevolent creativity: a malevolent idea journey
Source: Front Psychol. 2025 Dec 4;16:1695259. doi: 10.3389/fpsyg.2025.1695259 (PMC12712856; doi:10.3389/fpsyg.2025.1695259)
Supplement: Supplementary file 1 [file Supplementary_file_1.docx]

**Supplementary Materials to Manuscript**

**„Generating, evaluating, endorsing, and implementing malevolent creativity: A malevolent idea journey”**

**Part A – Idea Forecasting**

***Participants and Procedure***

N = 160 participants (84 women, 76 men; *M*age = 25.11, *SD* = 7.91) took part in this study, whose primary findings are included in another submitted manuscript (exploring links between true crime consumption and malevolent creativity). All participants gave informed consent to participate in the online study (implemented via LimeSurvey), which lasted ~1.5 hours in total. The study (as part of a larger research project) was approved by the authorized ethics committee of the University of Graz, Austria (GZ. 39/4/63 ex 2022/23).

***Test Materials***

Participants filled in various self-report questionnaires, including the short version of the **Buss-Perry Aggression Questionnaire** (BPAQ, Bryant & Smith, 2001), with includes three items each for physical aggression, verbal aggression, hostility, and anger (rated on a scale from 1 = does not apply to 4 = strongly applies). Items were summed up into a composite score for trait aggression (*M* = 18.84, *SD* = 4.75, *Min* = 12, *Max* = 34, Cronbach *α* = .79).

They also filled in the **Narcissistic Rivalry and Admiration Scale** (NARQ, short version; Leckelt et al., 2018), which includes six items (e.g., “*Being a very special person gives me a lot of strength*”), rated on a scale from 1 = not agree at all to 6 = agree completely. Items were summed up into a composite score for trait narcissism (*M* = 13.57, *SD* = 4.87, *Min* = 6, *Max* = 27, Cronbach *α* = .76).

Subsequently, participants were presented with two items from the **Malevolent Creativity Test** (for full description, see Perchtold-Stefan et al., 2021ab, 2022abcd, 2023) and were given three minutes per item to generate as many creative ideas to take revenge on a wrongdoer (situation 1: an inconsiderate colleague spilling coffee on one’s favorite book; situation 2: an unfair neighbor withholding money despite promising it for one’s help in renovating their flat). Answers were rated for fluency (number of instruction-conforming ideas, one experienced rater; *M* = 4.24, *SD* = 2.17), malevolence of ideas (rated from 1 = minimally malevolent to 6 = highly malevolent; 4 raters, ICC = .95; *M* = 2.20, *SD* = 0.40), and originality of ideas (rated from 1 = not original to 6 = highly original; 4 raters, ICC = .93; *M* = 2.24, *SD* = 0.63). A composite score of total malevolent creativity was computed, indicating the number of ideas that were at least moderately original (≥3; *M* = 2.34, *SD* = 2.62).

Afterwards, participants were asked to **forecast under which circumstance** they would most likely implement their generated ideas in real-life, and rated ten options on a scale from 1 (would very likely not implement to 5 (would very likely implement). Options (e.g., “*wanting to restore justice*”) were selected based on themes in previous malevolent creativity tests (see Perchtol-Stefan et al., 2022). For the full item list, see Table 2.

***Statistical Analysis and Results***

A repeated measures ANOVA was run using the within factor “forecasted circumstance” to test for differences in most and least likely circumstances for malevolent creativity implemented. Subsequently, forecasted circumstances were correlated with malevolent creativity indices (fluency, malevolence, originality, and total malevolent creativity) as well as trait-aggression to test whether people high in malevolent creativity and trait aggression was linked to forecasting certain circumstances more than others (Pearson’s correlations).

The repeated measures ANOVA yielded a significant effect of “forecasted circumstance” (*F*9,151 = 39.97, *p* < .001; *ηp²* = 0.70). Participants forecasted highest likelihood for idea implementation if *friends or family members were hurt by the wrongdoers’ actions* (*M* = 4.11, *SD* = 1.03), followed by the *wrongdoer not showing remorse for their behavior* (*M* = 3.71, *SD* = 1.13), and wanting to *restore justice* (*M* = 3.42, *SD* = 1.18). Conversely, the *situation threating one’s world view* (*M* = 2.42, *SD* = 1.14), *distance to the wrongdoer* (*M* = 2.54, *SD* = 1.26) and *other people witnessing the situation* (*M* = 2.61, *SD* = 1.23) received the lowest ratings. See Table 2 for detailed descriptive statistics.

Participants higher in malevolent creativity more likely forecasted implementation of their harmful creative ideas if they were sure *there would be no negative consequences* (total malevolent creativity: *r* = .17, *p* = .029), for *wanting to restore justice* (*r* = .18, *p* = .020) and if the *targeted person was not close to them* (*r* = .21, *p* = .007). Conversely, participants higher in trait aggression and narcissism most likely forecasted implementation of their harmful creative ideas if they felt *high anger* (aggression: *r* = .24; *p* = .003; narcissism: *r* = .15, *p* = .056), if *other people witnessed the situation* (aggression: *r* = .23, *p* = .004; narcissism: *r* = .18, *p* = .023), or if they *were hurt in their pride* (aggression: *r* = .22, *p* = .006; narcissism: *r* = .025, *p* = .001). See Table 3 for full correlational matrix.

**Table 2.** Descriptive statistics for forecasted circumstances of malevolent idea implementation

|  | Min | Max | Mean | SD |
| --- | --- | --- | --- | --- |
| The person does not show the slightest remorse for their actions | 1 | 5 | 3.71 | 1.13 |
| I am hurt in my pride | 1 | 5 | 2.99 | 1.28 |
| Other people are witnessing the situation | 1 | 5 | 2.61 | 1.23 |
| It all depends on how angry I am feeling in the moment | 1 | 5 | 3.02 | 1.27 |
| I am incurring financial damage | 1 | 5 | 3.05 | 1.26 |
| My entire worldview changes | 1 | 5 | 2.43 | 1.14 |
| I want to restore justice | 1 | 5 | 3.42 | 1.18 |
| Not only I am affected, but also my family and friends | 1 | 5 | 4.11 | 1.03 |
| The person is not close to me | 1 | 5 | 2.54 | 1.26 |
| I am sure that there will be no negative consequences for me | 1 | 5 | 3.03 | 1.38 |

**Note.** n = 160. Min = Minimum, Max = Maximum, SD = Standard deviation

**Table 3.** Correlation matrix of forecasted implementation with malevolent creativity, aggression, and narcissism

|  |  | 1 | 2 | 3 | 4 | 5 | 6 | 7 | 8 | 9 | 10 | 11 | 12 | 13 | 14 | 15 | 16 |
| --- | --- | --- | --- | --- | --- | --- | --- | --- | --- | --- | --- | --- | --- | --- | --- | --- | --- |
| 1 | MCT Total | -- |  |  |  |  |  |  |  |  |  |  |  |  |  |  |  |
| 2 | MCT Fluency | .63^**^ | -- |  |  |  |  |  |  |  |  |  |  |  |  |  |  |
| 3 | MCT Malevolence | .49^**^ | .36^**^ | -- |  |  |  |  |  |  |  |  |  |  |  |  |  |
| 4 | MCT Originality | .76^**^ | .35^**^ | .68^**^ | -- |  |  |  |  |  |  |  |  |  |  |  |  |
| 5 | Aggression | .11 | .08 | .13 | .05 | -- |  |  |  |  |  |  |  |  |  |  |  |
| 6 | Narcissism | .10 | .12 | .16^*^ | .08 | .41^**^ | -- |  |  |  |  |  |  |  |  |  |  |
| 7 | The person does not show remorse for their actions | .16^*^ | .11 | .09 | .13 | .10 | .00 | -- |  |  |  |  |  |  |  |  |  |
| 8 | I am hurt in my pride | .06 | .03 | -.01 | .08 | .22^**^ | .25^**^ | .39^**^ | -- |  |  |  |  |  |  |  |  |
| 9 | Other people are witnessing the situation | .06 | .01 | -.05 | .10 | .23^**^ | .18^*^ | .29^**^ | .48^**^ | -- |  |  |  |  |  |  |  |
| 10 | It all depends on how angry I am feeling in the moment | .11 | .18^*^ | .12 | .08 | .24^**^ | .15 | .33^**^ | .27^**^ | .30^**^ | -- |  |  |  |  |  |  |
| 11 | I am incurring financial damage | .00 | .05 | -.06 | -.08 | .11 | .13 | .37^**^ | .37^**^ | .34^**^ | .26^**^ | -- |  |  |  |  |  |
| 12 | My entire worldview changes | .14 | .07 | .02 | .08 | .09 | .04 | .19^*^ | .24^**^ | .19^*^ | .16^*^ | .39^**^ | -- |  |  |  |  |
| 13 | I want to restore justice | .18^*^ | .17^*^ | .17^*^ | .15 | .12 | .03 | .36^**^ | .19^*^ | 0.10 | .25^**^ | .26^**^ | .32^**^ | -- |  |  |  |
| 14 | Not only I am affected, but also my family and friends | .08 | .03 | .10 | .11 | .05 | .08 | .35^**^ | .16^*^ | .23^**^ | .19^*^ | .27^**^ | .17^*^ | .33^**^ | -- |  |  |
| 15 | The person is not close to me | .21^**^ | .14 | .20^*^ | .27^**^ | .18^*^ | .14 | .37^**^ | .32^**^ | .22^**^ | .22^**^ | .24^**^ | .24^**^ | .19^*^ | .20^*^ | -- |  |
| 16 | I am sure that there will be no negative consequences for me | .17^*^ | .21^**^ | .16^*^ | .22^**^ | .20^*^ | .23^**^ | .29^**^ | .41^**^ | .30^**^ | .24^**^ | .33^**^ | .15 | .24^**^ | .15 | .36^**^ | -- |

**Note.** ** p < .01, * p < .05

**Part B – Malevolent Creativity Endorsement**

***Participants and Procedure***

In total, *n* = 21 participants (all students) took part in the experiment. Due to the nature of the pilot study, demographics were not assessed. All participants gave informed consent to participate in an online survey (hosted via LimeSurvey) on their perception of aggressive behaviors (~ 45 min). The study (as part of a larger research project) was approved by the authorized ethics committee of the University of Graz, Austria (GZ. 39/47/63 ex 2024/25).

***Test Materials***

For the purpose of this study, 46 item pairs reflecting aggressive behaviors were created (92 items total). Each item pair represented the same aggressive behavior, but once phrased as ordinary, uncreative aggression (e.g., *tripping somebody up on their way to work*) and once phrased as creative aggression (e.g., *paying a police officer to trip somebody up on their way to work*). Creative versions of the aggressive behaviors were created based on participants’ previous answers on a malevolent creativity test (Perchtold-Stefan et al, 2021ab; 2022abcd) and by brainstorming in teams. Items reflected different categories of aggression one may encounter in real-life (physical aggression, property aggression, social aggression, verbal threats and tricks/pranks, see Perchtold-Stefan et al., 2023). Participants rated each item according to….

1. Physical Harmfulness: How harmful is this behavior on a physical level?
   1 = not harmful at all to 6 = extremely harmful
2. Mental/psychological harmfulness: How harmful is this behavior on a mental/psychological level? 1 = not harmful at all to 6 = extremely harmful
3. Short-term Consequences: How bad would the consequences be for the targeted person in the short-term? 1 = not bad at all to 6 = extremely bad
4. Long-term Consequences: How bad would the consequences be for the targeted person long-term? 1 = not bad at all to 6 = extremely bad
5. Creativity: How original/creative is this behavior?
   1 = not creative at all to 6 = extremely creative
6. Amusement: How funny is this behavior?
   1 = not funny at all to 6 = extremely funny
7. Surprise How surprising/unexpected is this behavior?
   1 = not surprising at all to 6 = extremely surprising
8. Endorsement (general): If somebody used this type of behavior to take revenge on an unfair/mean person, would you endorse it? 1 = not at all to 6 = definitely
9. Liking: If you saw this behavior on social media, would you like it?
   1 = would definitely not like to 6 = would definitely like
10. Sharing: If you saw this behavior on social media, would you share it with your friends?
    1 = would definitely not share to 6 = would definitely share

At the beginning of the online survey, participants were presented with the following instruction: *“Sometimes in daily life, people treat us unfairly, are rude to us, or intentionally cause us harm. These people could be friends, colleagues, acquaintances, etc. In this survey, you will be presented with examples of behaviors that people could use to take revenge or punish these wrongdoers. You will be asked to rate each behavior for aspects like harmfulness, uniqueness, or consequences. Please answer as honestly as possible how you would judge these behaviors. Some behaviors will appear rather similar to you, but please rate each of them on their own.”*

Subsequently, all items were presented in randomized order to participants.

***Statistical Analysis and Results***

Item ratings were averaged to yield mean scores for ordinary aggression vs. creative aggression ratings. Subsequently, ten paired sample t-tests were run to test for basic rating differences between ordinary and creative aggression.

As a manipulation check, creative aggression was rated as significantly more creative than ordinary aggression (*t*20 = -12.95, *p* < .001, *d* = -2.83). There were no differences in ratings of ordinary vs. creative physical aggression (*t*20 = 0.33, *p* = .743, *d* = 0.07), in ratings of ordinary vs. creative mental aggression (*t*20 = 0.38, *p* = .706, *d* = 0.08), or in ratings of long-term consequences of ordinary vs. creative aggression (*t*20 = -0.51, *p* = .618, *d* = -0.11). However, short-term consequences were rated as less severe for creative aggression than for ordinary aggression (*t*20 = 2.50, *p* = .021, *d* = 0.55). Additionally, creative aggression was perceived as more surprising (*t*20 = -8.33, *p* <.001, *d* = -1.81), and more humorous (*t*20 = 8.21, *p* <.001, *d* = -1.79) and received more endorsement than ordinary aggression (*t*20 = -2.54, *p* = .019, *d* = -0.56). Lastly, participants were more likely to like (*t*20 = -5.56, *p* < .001, *d* = -1.21) and share creative aggression (*t*20 = -6.21, *p* < .001, *d* = -1.35) on social media compared to ordinary aggression. See Table 1 for a summary including descriptive statistics.

**Table 1.** Descriptive statistics and paired-sample t-tests results for aggression pairs

|  | Min | Max | Mean | SD | t_20_ | | p |
| --- | --- | --- | --- | --- | --- | --- | --- |
| Ordinary aggression creativity | 1.04 | 3.36 | 2.057 | 0.60 | -12.94 | <.001 | |
| Creative aggression creativity | 1.15 | 4.67 | 3.63 | 0.89 |  |  |  |
| Ordinary aggression physical harm | 1.52 | 5.35 | 2.59 | 1.01 | 0.33 | | .743 |
| Creative aggression physical harm | 1.61 | 5.37 | 2.57 | 0.94 |  |  |  |
| Ordinary aggression mental harm | 2.39 | 5.65 | 3.71 | 1.01 | 0.38 | | .706 |
| Creative aggression mental harm | 2.37 | 5.65 | 3.68 | 0.93 |  |  |  |
| Ordinary aggression short-term consequences | 3.04 | 5.80 | 4.18 | 0.79 | 2.50 | | .021 |
| Creative aggression short-term consequences | 3.07 | 5.74 | 4.06 | 0.72 |  |  |  |
| Ordinary aggression long-term consequences | 1.93 | 5.22 | 3.03 | 0.96 | -0.51 | | .618 |
| Creative aggression long-term consequences | 1.96 | 5.22 | 3.06 | 0.87 |  |  |  |
| Ordinary aggression funniness | 1.02 | 2.35 | 1.51 | 0.41 | -8.21 | | <.001 |
| Creative aggression funniness | 1.07 | 4.33 | 2.46 | 0.82 |  |  |  |
| Ordinary aggression surprise | 1.07 | 4.04 | 2.49 | 0.76 | -8.33 | | <.001 |
| Creative aggression surprise | 1.30 | 4.80 | 3.60 | 0.94 |  |  |  |
| Ordinary aggression endorsement | 1.00 | 5.63 | 1.84 | 1.05 | -2.54 | | .019 |
| Creative aggression endorsement | 1.00 | 5.74 | 1.95 | 1.06 |  |  |  |
| Ordinary aggression liking | 1.00 | 2.83 | 1.53 | 0.59 | -5.56 | | <.001 |
| Creative aggression liking | 1.00 | 3.96 | 2.01 | 0.78 |  |  |  |
| Ordinary aggression sharing | 1.00 | 3.03 | 1.71 | 0.66 | -6.21 | | <.001 |
| Creative aggression sharing | 1.00 | 3.85 | 2.29 | 0.91 |  |  |  |

**Note.** n = 21. Min = Minimum, Max = Maximum, SD = Standard deviation, t = paired-sample t-test statistics, p = p-value

**Part C – Idea Selection 1**

***Participants and Procedure***

*Study 1*

N = 107 participants (58 women, 49 men; *M*age = 22.64, *SD* = 4.52) took part in this study, whose primary findings were published in Perchtold-Stefan et al. (2021a). All participants gave informed consent to participate in an in-person testing session at the Department of Psychology, University of Graz (~ 120 min). The study was approved by the authorized ethics committee of the University of Graz, Austria (GZ. 39/69/63 ex 2017/18).

*Study 2*

N = 74 participants (all women; *M*age = 24.14, *SD* = 4.46) took part in this study, whose primary findings were published in Perchtold-Stefan et al. (2021b). All participants gave informed consent to participate in an in-person testing session at the Department of Psychology, University of Graz (~ 150 min). The study was approved by the authorized ethics committee of the University of Graz, Austria (GZ. 39/48/63 ex 2018/19).

***Test Materials***

In both studies, participants completed the **Malevolent Creativity Test** (4 item version; for full description, see Perchtold-Stefan et al., 2021ab, 2022abcd, 2023) and were given three minutes per item to generate as many creative ideas to take revenge on a wrongdoer. Answers were rated for fluency, malevolence (two experienced raters) as well as malevolence of ideas (rated from 1 = minimally malevolent to 4 = highly malevolent; 4 raters), and originality of ideas (rated from 1 = not original to 4 = highly original; 4 raters). A composite score of total malevolent creativity was computed, indicating the number of ideas that were at least moderately original (≥2). See Table 4 for descriptive statistics of MCT scores in both studies.

Afterwards, participants were asked to indicate as a dichotomous choice, whether they would more likely not (0) or more likely (1) implement their generated ideas in real-life.

***Statistical Analysis and Results***

Descriptive statistics were computed for implementation choice of generated MCT ideas (yes/no). For study 1, independent sample t-tests were run to test for differences in malevolent creativity indices between implementation groups. For study 2, comparisons could not be conducted due to highly unequal group sizes.

In study 1, 81.3 % of participants indicated that they would likely not implement their generated malevolent creativity ideas in real-life, while 18.7% indicated that they more likely would implement their ideas. In study 2,85.9% of participants indicated “likely no” for implementation, while only 7.1 % indicated “likely yes”).

In study 1, significant differences in malevolent creativity were observed between the implementation groups. Participants indicating that they would more likely implement their generated ideas in real-life showed lower ideational fluency (*t*105 = 3.96; *p* <.001, *d* = .98), lower malevolence (*t*105 = 5.28 *p* <.001, *d* = 1.03), lower originality (*t*105 = 4.60, *p* <.001, *d* = 1.14) and lower total malevolent creativity of ideas (*t*105 = 3.04, *p* = .003, *d* = 0.76). Also see Table 4.

**Table 4.** Descriptive statistics and t-test statistics for malevolent creativity indices

|  | Min | Max | Mean | SD | ICC | t(p) |
| --- | --- | --- | --- | --- | --- | --- |
| **Study 1** (n = 107) |  |  |  |  |  |  |
| MCT Total | 0.00 | 17.00 | 5.40 | 4.40 | **--** | t105 = 3.04 (.003) |
| MCT Fluency | 1.00 | 36.00 | 16.42 | 7.51 | .99 | t105 = 3.96 (<.001) |
| MCT Malevolence | 1.00 | 2.96 | 2.15 | 0.35 | .88 | t105 = 5.28 (<.001) |
| MCT Originality | 1.00 | 3.13 | 1.80 | 0.38 | .91 | t105 = 4.60 (<.001) |
| **Study 2** (= 74) |  |  |  |  |  |  |
| MCT Total | 0.00 | 6.50 | 2.30 | 1.29 | **--** | **--** |
| MCT Fluency | 1.00 | 33.00 | 16.19 | 5.57 | .99 | **--** |
| MCT Malevolence | 1.59 | 2.66 | 2.12 | 0.25 | .81 | **--** |
| MCT Originality | 1.35 | 3.09 | 2.06 | 0.35 | .91 | **--** |

**Note.** Min = Minimum, Max = Maximum, SD = Standard deviation, t = independent sample t-test statistics for implementation (yes/no), p = p-value

**Part D – Idea Selection 2**

***Participants and Procedure***

The same as for Part C – study 1 (primary findings published in Perchtold-Stefan et al., 2021a).

***Test Materials***

Participants filled in various questionnaires, including the Comprehensive Misconduct Inventory (Williams et al., 2007), which assesses one’s propensity to engage in misconduct behaviors (e.g., driving misbehavior) with 58 items. Participants are asked to indicate how many times they engaged in a certain behavior during adolescence (high school) and in the past month. Overall misconduct is measured with seven subscales: soft drug abuse, hard drug abuse, minor criminality, serious criminality, driving misbehavior, bullying/harassing, and anti-authority misbehavior, which are then averaged into a general misconduct factor. Prior to averaging, all items were standardized. In the present study, the two subscales for criminality were also averaged into a general criminality index, the two subscales for drug abuse were averaged into a general drug abuse index, and an additional index of current misconduct behavior was computed (items addressing the past month). See Table 5 for descriptive statistics prior to standardizing.

**Table 5.** Descriptive statistics of self-reported misconduct behavior

|  | Min | Max | Mean | SD | α |
| --- | --- | --- | --- | --- | --- |
| Soft drug abuse | 0.00 | 165.25 | 10.46 | 25.53 | .67 |
| Hard drug abuse | 0.00 | 3.50 | 0.21 | 0.71 | .70 |
| Minor criminality | 0.00 | 950.00 | 21.32 | 93.65 | .39 |
| Serious criminality | 0.00 | 200.00 | 2.79 | 19.33 | .48 |
| Driving misbehavior | 0.00 | 6.00 | 0.45 | 0.90 | .54 |
| Bullying/harrasing | 0.00 | 140.00 | 3.60 | 18.17 | .38 |
| Anti-authority behavior | 0.00 | 134.00 | 9.85 | 22.83 | .52 |
| General substance abuse | 0.00 | 2.68 | 0.00 | 0.52 | .76 |
| General criminality | 0.00 | 1.29 | 0.00 | 0.33 | .49 |
| High school misbehavior | 0.14 | 223.37 | 9.71 | 24.62 | .79 |
| Current misbehavior | 0.00 | 9.93 | 1.39 | 1.97 | .57 |

**Note.** Min = Minimum, Max = Maximum, SD = Standard deviation, α = Cronbach’s alpha: items 2, 19, 33, and 38 were removed from the analysis as all participants had indicated zero misbehaviors.

**Malevolent Creativity Test:** the same as for Part C – study 1

***Statistical Analysis and Results***

Pearsons’ correlations were computed to map relationships between malevolent creativity indices and various aspects of self-reported misconduct. Note that most Cronbach alphas are to be interpreted as poor, which questions the validity of the scales.

There were no significant correlations between total malevolent creativity and any of the misbehavior indices, although links to general criminality showed a trend level association (*r* = .18, *p* = .072). No significant relationships were observed for MCT fluency either (see Table 6). General criminality (r = .24), hard drug abuse (*r* = .19), general substance abuse (*r* = .22) and high school misbehavior (*r* = .20) positively correlated with MCT malevolence. Only general criminality positively correlated with MCT originality (*r* = .21), although high school misbehavior also showed a trend level association (*r* = .18, *p* = .067).

**Table 6.** Correlation matrix for links of malevolent creativity indices with misconduct behavior

|  |  | 1 | 2 | 3 | 4 | 5 | 6 | 7 | 8 | 9 | 10 | 11 | 12 | 13 | 14 | 15 |
| --- | --- | --- | --- | --- | --- | --- | --- | --- | --- | --- | --- | --- | --- | --- | --- | --- |
| 1 | MCT Total | -- |  |  |  |  |  |  |  |  |  |  |  |  |  |  |
| 2 | MCT Fluency | .66^**^ | -- |  |  |  |  |  |  |  |  |  |  |  |  |  |
| 3 | MCT Malevolence | .55^**^ | .42^**^ | -- |  |  |  |  |  |  |  |  |  |  |  |  |
| 4 | MCT Originality | .71^**^ | .24^*^ | .62^**^ | -- |  |  |  |  |  |  |  |  |  |  |  |
| 5 | Minor Criminality | .14 | .12 | .19^*^ | .16 | -- |  |  |  |  |  |  |  |  |  |  |
| 6 | Serious Criminality | .12 | .03 | .17 | .16 | .13 | -- |  |  |  |  |  |  |  |  |  |
| 7 | General Criminality | .18 | .10 | .24^*^ | .21^*^ | .68^**^ | .81^**^ | -- |  |  |  |  |  |  |  |  |
| 8 | Soft Drug Abuse | .09 | .04 | .17 | .14 | .57^**^ | .15 | .45^**^ | -- |  |  |  |  |  |  |  |
| 9 | Hard Drug Abuse | .01 | .05 | .19^*^ | .06 | .25^**^ | .28^**^ | .35^**^ | .44^**^ | -- |  |  |  |  |  |  |
| 10 | General Substance Abuse | .06 | .05 | .22^*^ | .12 | .47^**^ | .26^**^ | .47^**^ | .81^**^ | .88^**^ | -- |  |  |  |  |  |
| 11 | Driving Misbehavior | .02 | .05 | .03 | -.07 | .25^**^ | .43^**^ | .46^**^ | .29^**^ | .15 | .25^**^ | -- |  |  |  |  |
| 12 | Bullying/Harassing | .14 | .12 | .08 | .16 | .27^**^ | .53^**^ | .55^**^ | .34^**^ | .05 | .21^*^ | .17 | -- |  |  |  |
| 13 | Anti-Authority Misbehavior | -.02 | -.06 | .01 | -.02 | .17 | .17 | .23^*^ | .13 | .04 | .10 | .38^**^ | .25^**^ | -- |  |  |
| 14 | High School Misbehavior | .11 | .05 | .20^*^ | .18 | .68^**^ | .61^**^ | .85^**^ | .73^**^ | .57^**^ | .76^**^ | .56^**^ | .52^**^ | .38^**^ | -- |  |
| 15 | Current Misbehavior | .10 | .12 | .13 | -.01 | .30^**^ | .34^**^ | .43^**^ | .40^**^ | .14 | .31^**^ | .53^**^ | .58^**^ | .63^**^ | .45^**^ | -- |

**Note.** ** p < .01, * p < .05
